# (Tele)Work and Care during Lockdown: Labour and Socio-Familial Restructuring in Times of COVID-19

**DOI:** 10.3390/ijerph182212087

**Published:** 2021-11-17

**Authors:** Iduzki Soubelet-Fagoaga, Maitane Arnoso-Martínez, Itziar Guerendiain-Gabás, Edurne Martínez-Moreno, Garbiñe Ortiz

**Affiliations:** Department of Social Psychology, University of the Basque Country, 20018 Donostia, Spain; maitane.arnoso@ehu.eus (M.A.-M.); itziar.guerendiain@ehu.eus (I.G.-G.); edurne.martinez@ehu.eus (E.M.-M.); garbine.ortiz@ehu.eus (G.O.)

**Keywords:** lockdown, inequality, wellbeing, on-site working, teleworking, gender, caregiving, work-life balance

## Abstract

COVID-19, and the lockdown requirement, altered our daily lives, including the restructuring of work and socio-familial organisation of millions of people. Through two studies, we explored how workers experienced this period. The first, qualitative study (N = 30) aimed to understand how workers lived through lockdown by identifying the key elements that shaped their experiences. Thematic content analysis revealed four emerging themes: (1) work and socio-health situation in which lockdown was experienced; (2) consequences on work organisation and resources available for change; (3) work–life balance management; and (4) psychosocial consequences and coping with the situation. The second, quantitative study (N = 332) explored the socio-health situation, new work organisation, work–life balance, and psychosocial consequences and coping strategies developed during this period, analysing participants’ differences in terms of gender, working modality (on-site or teleworking) and care responsibilities through ANOVA analysis. Results revealed the non-democratic nature of the pandemic, with differences and similarities according to gender, working modality and having or not having dependents. Results are discussed identifying areas that need to be addressed to ensure the well-being of workers.

## 1. Introduction

### 1.1. COVID-19: Restructuring Working Activity in a Context of Fear and Insecurity

COVID-19 arrived in early 2020 in a sudden way, disrupting daily routines and affecting mental and physical health. Specifically, COVID-19 has caused the infection of millions of people and the spread of anxiety, fear and stress [1]. In addition, the lockdown situation caused by the pandemic disrupted people’s lifestyles and the economy, leading to the restructuring of the working activities of millions of people [1]. The state of alarm decreed in Spain on March 15th, and the demand for lockdown in the following months, forced thousands of workers to continue, where possible, their work activities through teleworking, with only those considered essential workers being allowed to attend their jobs on-site. In Spain, 66% of workers continued to attend their jobs on-site, while 34% did so through telework [2].

Among the latter, the Labour Force Survey [3] revealed that, in the second quarter of the year, Spanish wage earners worked 3,798,700 h a week for free, with overtime hours worked (and not paid) soaring, surely because of this process of change and adaptation. Similarly, the study by Ruiz-Frutos and colleagues [4] showed how the workload of workers increased during lockdown. This may have been due, among other things, to the demands of having to make a hasty transition to a new work modality for many, such as teleworking.

Precisely, in a context where only 4.8% teleworked on a regular basis before the pandemic [5], the implementation of telework may have been forced, without organisations or workers being able to develop adequate investment in training and equipment [6]. Furthermore, for the effective implementation of telework it would be essential for organisations to adopt a flexible organisational culture and decentralised structure [7]; which, at present, does not correspond to the human resources policies pursued in most organisations, calling into question the traditional inflexible methods of this area [8].

In addition, workers were exposed to a previously unknown virus during lockdown, which led to a fear of infection and illness that was experienced collectively as part of the community. In this regard, Mertens et al. [9] have shown the consequences of fear of infection and compulsive checking behaviours related to COVID-19, leading to constant rumination associated with the possibility of infection. 

COVID-19 fear of illness had particularly strong consequences (anxiety, depression) among people with previous mental illness [10,11]. In fact, psychological distress during lockdown was explained by mental health status prior to the pandemic, rather than by the type of professions people exercised (health workers or not) [10]. However, the above research did not examine the possibility that these impacts depended on work modality (telework or on-site work), which has been shown to be relevant in other research [4,12].

In fact, while the fear of infection was present in both on-site and teleworkers [4], it was higher among those who attended the workplace due to greater exposure to the virus [4,12], especially in the case of health workers [13]. In this respect, a recent study conducted in China during the pandemic context [14] reinforced the importance of organisational health measures (such as improvement of workplace hygiene or concerns on the health status of employees) in creating safe spaces for workers and predicting lower psychiatric symptomatology among them (including PTSD symptoms, stress, anxiety, depression, and insomnia).

Moreover, the fear of infecting relatives and loved ones was prominent, particularly among people with dependent children [15,16]. Gender roles may have also played a role in the fear of becoming infected or ill. This idea is supported by the Molero et al. study [15], which shows a greater concern of becoming infected in women. Similarly, Dryhurst et al. [17] found higher levels of risk perception in relation to COVID-19 among women, compared to men, in their research conducted in 10 countries across Europe, America and Asia. 

Furthermore, previous research on the psychosocial effects of pandemics has shown how concerns about the individual and societal economic consequences of such crises often predict the occurrence of stress among the population [18,19]. The Spanish state, the context in which this research takes place, is precisely one of the countries that has been hardest hit by the COVID-19 economic crisis [20], with a contraction of 18.5% in its gross domestic product (GDP) between April and June 2020. 

Finally, it should be borne in mind that the impact of economic crises is not the same for the entire population. Indeed, from a gender perspective, although outside the crisis context there are no gender differences in unemployment risk in developed countries, nor in the perception of this risk, it is expected that the pandemic’s economic consequences will be suffered more by women than by men [21]. Precisely, various reports point out how women will suffer the consequences of this economic crisis more severely, due to the feminisation of the sectors that will be most affected by it (commerce, tourism, and hostelry) [22]. In addition, perceived economic threat was a major concern during lockdown among families with dependent children [16].

### 1.2. Work-Life Balance in Times of Pandemic

It should be borne in mind that these changes in the work environment took place in a context of school and day-care closures, leaving childcare completely relegated to the private sphere [23]. Without additional resources for care, work–life balance took on significant relevance among those who continued working [24], and, because of the existing imbalance in care responsibilities, this demand seems to have fallen mainly on women [23].

Combining work and family life is not easy, even outside of an extraordinary situation such as a pandemic and a lockdown, and may explain the work–related stress suffered by workers [25,26]. Netemeyer et al. [27] defined two types of conflict resulting from this situation: work-family conflict, when work obligations impact on family care, and family-work conflict, when family responsibilities impact on work activity.

In relation to work modality (out of the pandemic context), research shows the advantages of teleworking compared to working on-site as a work-life balance measure. Teleworkers value positively the flexibility they have in organising their working time [28,29,30]. Thus, people who telework regularly tend to experience low work-family conflict [31,32]. Even so, several studies point out the difficulties created by the lack of separation or permeability between work and family life, reporting family disputes caused by poor boundaries (referring to both work-family and family-work conflict) [32,33,34]. The difficulties in carrying out work because of family interference are also highlighted, in the particular context of family-work conflict.

Precisely in the lockdown context, research published so far shows that typically negative aspects of telework have been accentuated. The difficulty in separating spaces has been more evident in the context of exceptionality experienced during lockdown [35]. In total, 44% of teleworkers in the Spanish state had difficulty concentrating on work because of family responsibilities, while more than 15% said that teleworking had prevented them from spending as much time with their family as they would have liked [36].

Moreover, from a gender perspective, and beyond the pandemic context, literature has shown that men who telework have less conflict in carrying out their professional activity in comparison to women, as teleworking does not seem to be a sufficient reason for increasing men’s involvement in domestic and care work [37]. Similarly, Hartig et al. [38] found that the home was a stress-reducing space for men, with men who telework having lower stress levels than those who do not; whereas, teleworking did not lead to the same decrease in stress levels for women, who actually experienced more stress than those women who work on-site. These results could be explained by different motivations for teleworking depending on gender, since while the main motive for women is to improve work-life balance and be able to exercise their role as carers; men tend to opt for teleworking for individual and professional motives [37]. 

In this regard, several studies point out the greater difficulties encountered by women in the practice of telework, as well as the danger that this work modality perpetuates and accentuates gender roles in women with care responsibilities, especially when gender is combined with other variables (such as having dependents) [37,38,39]. Thus, and within the framework of exceptionality of the lockdown measures, it is likely that this total relegation of care to the private sphere has created greater challenges for women’s living conditions, health and well-being [40,41]. In fact, Escuerdo-Castillo et al. [42] reported a worse self-perceived well-being among women compared to men during this period.

### 1.3. Maintaining Normality in Abnormal Contexts: How to Make It Happen?

Organizations’ concern to maintain work normality during lockdown and workers’ need to meet their superiors’ expectations of normality, may have led to dynamics where workers may have been forced to overwork. Indeed, this overwork situation led workers to be more stressed than usual [4]. The challenge of having to maintain a situation of normality could also have translated into a more work-oriented dynamic in people’s lives. This has been defined in the literature in terms of workaholism [43,44], as a compulsive internal drive to work excessively [44,45], giving more energy than required to work [45] and neglecting other aspects of life by being too involved in work [46]; which in turn generates increased work stress [47,48].

The likelihood of developing workaholic behaviour depends on many factors, such as psychological and personality variables, but it also responds to contextual and organisational factors, such as the pressure that the situation and organisations may place on such behaviour [49,50]. Moreover, telework has been identified as a significant factor in the development of addictive behaviours [51,52].

Despite its risks for workers’ health, it is true that workaholism can be studied in its more positive dimension [48], as a possibility of enjoyment at work through the ability to escape from everyday worries [45,50]. In this sense, dedication to work may have become a coping strategy in itself during lockdown, through which one could find a source to occupy his/her time and make the lockdown situation more bearable. Self-care recommendations during this context were common, both in the media and in specific guidelines that stressed the importance of having a healthy routine and developing other skills and hobbies to maintain a good state of health [53].

From a gender perspective, studies prior to the pandemic have found a greater tendency to use emotional coping strategies in women and more solution-focused coping strategies in men [54]. In summary, the concept of workaholism seems to be relevant for understanding the relationship people have had with work during the lockdown context, especially from a gender perspective. However, to our knowledge, no research has yet been performed that has taken this into account.

### 1.4. Aims of This Research

Through two consecutive studies, developed in an exploratory way and with a mixed methodology, we aimed to delve into the way in which workers dealt with their relationship with work in a context of exceptionality such as lockdown. Previous research has shown how lockdown affected workers’ mental health, economic uncertainty, and work–family conciliation (among others). However, no study to date has considered all these effects in an integrated manner, taking into account a range of structural and psychosocial variables that allow for a holistic understanding of this experience.

Thus, the first study, based on a qualitative approach, aimed to explore how workers experienced their work situation during this period through their own narratives, identifying the psychosocial processes people went through in trying to cope with the situation and maintain their health and well-being in an abnormal and stressful context. Few investigations have been carried out from this perspective during lockdown, so we sought to understand workers’ narratives and voices using semi-structured interviews.

The second study, through a structured quantitative survey, aimed to investigate the effect that key variables such as gender, work modality (on-site or telework) and having or not having dependents may have had on the way in which lockdown was experienced, as there are no studies that jointly analyse the influence of these variables on workers’ psychosocial situation. In particular, we will analyse how these variables influenced the lockdown experience, in relation to (1) adaptation to changes in work activity, (2) fear of contagion, (3) conflicts derived from work–life balance, and (4) psychosocial consequences and coping strategies. 

## 2. Study 1

### 2.1. Materials and Methods

#### 2.1.1. Participants

30 people were interviewed, of whom 19 were women and 11 were men (M_age_ = 39.8; SD = 1.84). A total of 11 respondents had dependents and 18 respondents had no dependents. In terms of employment status, 16 were teleworking, 7 were working on-site, 5 were in an ERTE (temporary employment regulation files implemented by the Spanish Government), and 2 had been unemployed since the lockdown began. The sample size criterion was based on discourse saturation. That is, recruitment was stopped once it was found that the new interviews did not introduce additional content to the previous interviews conducted [55].

#### 2.1.2. Procedure

The interviews were conducted between April and May 2020. Participants were recruited by an online advertisement, spread through social networks (WhatsApp, Facebook) (Meta Platforms, Inc., Menlo Park, CA, USA) Twitter (Twiter, San Francisco, CA, USA), requesting participation in a study on labour consequences of lockdown promoted by the University of the Basque Country. A telephone or Skype (Microsoft, Luxembourg, Luxembourg) interview was arranged with those who agreed to participate, with an average duration of 45 min.

#### 2.1.3. Instrument

The interviews were conducted in a semi-structured way, and started by explaining to participants the study objectives and interview structure. We then asked them to provide us with some specific data to identify different profiles: age, gender identity, work field, whether they had dependents, their employment situation during lockdown (working, unemployed, with a reduction in activity, etc.) and their work modality (on-site, teleworking). 

Once these data were registered, we asked participants to tell us more deeply about their work situation (e.g., “How are you currently performing your job in this lockdown context?”; “How has your job adapted to this new context?”; “How are you living this adaptation process?”). Some questions were also asked to explore the resources available to them in order to continue with work activity (e.g., “How has your organisation responded to these changes?”; “What resources have been made available from your organisation to continue with work activity?”). 

Subsequently, the interview sought to find out how participants were experiencing this situation emotionally, both in relation to COVID-19 and to adapting to work in these new circumstances (e.g., “How are you experiencing this situation?”; “How is your mental and emotional health in these circumstances?”; “Which aspects help you and which make it difficult for you to cope with this exceptional situation?”). Throughout the course of the interview, interviewers incorporated specific questions aimed at reflecting on how gender, care responsibilities, and work–family conciliation in general were affecting this process (e.g., “How do you live under these exceptional circumstances with regard to work–life balance?”). 

The interview ended when the participants did not offer any additional content and the discourse seemed saturated. Interviewers thanked participants for their time and the value of their testimonies, and asked them to provide their e-mail addresses so that they could be informed of research results.

#### 2.1.4. Data Analysis 

The interviews were recorded and transcribed verbatim, resulting in a volume of 29,437 words, which were subjected to a thematic analysis aimed at broadening the interpretative dimension of the phenomenon studied. For the thematic analysis, researchers read the transcripts repeatedly. With the research objective in mind, they proceeded to select the parts of the text that were significant in answering the research question. These parts of the text were assigned a label capturing a more general meaning. As researchers discussed the nature of the codes, the information began to be articulated in a theoretical way, drawing on previous theories and studies on the same topic.

One aspect that adds validity to this study is that all the codes created from the texts were collected by the proposed themes. Each part of the text was exclusively included in one code, and each code in a single category. Standard procedures were used to estimate the reliability of the analysis using inter-judge reliability [56], exploring the coincidence in the number of codes and quotes assigned by each of the three researchers participating in this analysis. Finally, the agreement ratio was calculated by dividing the number of codes and the number of total citations (matching and non-matching), obtaining an acceptable ratio of 90% [57].

### 2.2. Results

Thematic analysis identified four general themes that answer the question of how people experienced their relationship with work during the lockdown period: (1) work and socio-health situation in which lockdown was experienced; (2) consequences on work organisation and resources available for change; (3) work–life balance management and centrality of life in the lockdown context, and (4) psychosocial consequences and coping with the situation. We present the main themes, sub-themes, codes and examples of statements from the qualitative analysis.

In relation to the work and socio-health situation of the people interviewed (see Table 1), testimonies give an account of the diversity of work situations that resulted from lockdown. While some were able to maintain their working hours and conditions, other interviews revealed the socio-economic vulnerability resulting from the state of alarm (with reductions in activity and wages, total cessation of activity or even dismissals, the latter being localised among those engaged in domestic work). This occurred in a context of dual fears, both in relation to the virus and the possibility of becoming infected, as well as in relation to the economic threat related to the announced economic crisis.

The second main theme detected concerned the consequences for work organisation and resources available for change (see Table 2). Interviews show a tendency amongst organisations to attempt to maintain normality in a context which was showing, day by day, the inevitability of accepting and coping with change, as well as a lack of capacity to respond to it. Interviews also reveal organisations’ mistrust of workers’ task commitment for telework; which contrasts with workers’ perception of increased responsibilities, that they had to assume to implement the changes and challenges the situation generated. Moreover, while some organisations, especially those where work involved face-to-face contact, put in place infection prevention measures; these resources did not reach the smallest companies or the most vulnerable employment niches. Regarding material resources provided for teleworking, interviews show that, in general, workers used their own resources (computers, connection, etc.), with organisational resources being reduced to the provision of platforms for teleworking and a limited training in their use.

The third theme raised issues related to work–life balance management and the dichotomy between work and life centrality, which led to an overlap between these two areas (see Table 3). Testimonies report difficulties arising from family-work conflict (as an expression of difficulties in fulfilling work tasks because of the need to care for dependents), but also difficulties arising from work-family conflict (expressed as the difficulty that work poses for the proper care of dependent children). The importance of having support to be able to carry out work-life balance is highlighted, as well as the fact that it has been a challenge that has particularly affected women, who have had to bear the burden of double working hours without the necessary resources to do so. Thus, interviews emphasised how lockdown has served to highlight the unsustainability of a system with serious difficulties in harmonising work and life.

For people without dependent children, a greater dedication to work could be experienced as a way of occupying their time. Those who saw their work activity diminished or paralysed said that they were able to rest and rediscover life; revealing that, in contexts of normality, work usually dominates workers’ lifetime. In any case, most of the interviews showed extended working hours and longer working days than usual; indicating that, in general, lockdown was dominated by the centrality of work in people’s lives.

Finally, the fourth theme, referring to the psychosocial consequences and coping with the situation, emphasised the isolation in which interviewees experienced their relationship with work, with a lack of ventilation spaces, or spaces for mutual support among colleagues (in face-to-face activity) (see Table 4). The exhaustion produced by the use or abuse of technological resources is also highlighted, as well as an increase in physical and psychological exhaustion because of efforts to adapt to change without sufficient resources and support. Thus, testimonies were collected with a high expression of overload, anxiety, stress, or burnout experiences among the people interviewed. Among the ones who were able to cope healthily with the situation, the establishment of routines, the possibility of maintaining a balance between the time dedicated to work and the time dedicated to life, as well as the ability to organise oneself, emerged as the most adaptive strategies in the face of this situation.

## 3. Study 2

### 3.1. Materials and Methods

#### 3.1.1. Participants

A total of 332 people participated (M_age_ = 42.32; SD = 10.19), of whom 178 were teleworking (65% women and 35% men; 20.3% with dependents and 79.7% without) and 154 were working on-site (51.5% women and 48.5% men; 21% with dependents and 79% without).

#### 3.1.2. Procedure

The sample of participants was recruited through an online questionnaire (survey-monkey platform) published on social networks (Facebook, WhatsApp, Twitter). Data collection was carried out during the state of alarm and lockdown period imposed by the Spanish Government because of the COVID-19 pandemic (10 April–10 May). Participants were informed of the purpose of the study, asked for permission to use their data, and were assured of anonymity and confidentiality.

#### 3.1.3. Instruments

Teleworkers and on-site workers answered the same questions, except for those related to organisational resources for task performance, where specific questions adapted to each work modality were used. 

##### Socio-Demographic Variables

The following variables were included: gender (1 = male, 2 = female), age and having or not having dependents (1 = no, 2 = yes). We also measured work modality through a question (“What type of employment are you currently in?”) with two response options (1 = going to my workplace; 2 = teleworking).

##### Socio-Health Situation

Fear of contagion. Measured through a question (“Indicate your agreement or disagreement with the following statements regarding the current situation triggered by the COVID-19 pandemic”) with 2 items created ad hoc (“I am afraid I might get sick from COVID-19”; “I am afraid of infecting my family/environment with COVID-19”) (α= 0.78) (r = 0.60, *p* < 0.01), on a Likert-type scale ranging from 1 = totally disagree to 7 = totally agree.

Perceived economic threat. Measured through a question (“Indicate your agreement or disagreement with the following statements regarding the current situation triggered by the COVID-19 pandemic”) with 4 items created ad hoc (“I am worried that I will have difficulty making ends meet because of this pandemic”; “I am concerned, because of this pandemic, that I will have a hard time making ends meet”; “I am afraid of the economic crisis that this pandemic will cause”; “I am afraid that this crisis will aggravate social inequality in our society”) (α = 0.70), on a Likert-type scale ranging from 1 = totally disagree to 7 = totally agree.

##### New Work Organisation

Hours worked. Measured through a question (“How many hours do you work underthese new conditions?”) with three response options (1 = less than usual, 2 = the same, 3 = more than usual).

Organisational resources provided by organisations for teleworking. Measured through a question (“How do you rate the various services that the organisation you work for has made available for you to continue working from home?”) with 7 items created ad hoc (“training”, “organisational computer”; “technical advice”; “corporate phone”; “licences for access to platforms for video-conferencing or collaborative settings”, “grants to pay for internet connection”, “grants to pay electricity bills”) (α = 0.80), on a Likert-type scale ranging from 1 = non-existent to 7 = totally adequate.

Organisation’s ability/readiness to telework. Measured through an ad hoc question of 1 item (“To what extent do you feel that your organisation was prepared for this way of working?”), on a Likert-type scale ranging from 1 = not at all to 7 = completely.

Safety at on-site work. Measured through 1 item generated ad hoc (“I feel safe going to my workplace”), on a Likert-type scale ranging from 1 = not at all to 7 = completely.

Productivity control measures. Measured through 1 item (“During this period, do you feel that your company/organisation has introduced new mechanisms to monitor your production while you are teleworking?”) with two response options (1 = yes; 2 = no).

##### Conciliation 

Work-family and family–work conflict [27]. It consists of two scales: the first one explores work-family conflict through 3 items (“My work obligations interfere with my family life”; “The time demands of my job make it difficult for me to assume my family responsibilities”; “The pressure I am under at work makes it difficult for me to take on my family responsibilities”) (α = 0.91); while the second one examines family-work conflict through another 3 items (“My family or partner’s demands interfere with my workday”; “I have to stop doing things at work because of the demands of my family or partner”; “My family hinders my responsibilities at work, the time I need to spend at work or the fulfilment of my daily work obligations”) (α = 0.91). All items were measured through the following question: “Please indicate how strongly you agree with the following statements”, on a Likert-type scale ranging from 1 = totally disagree to 7 = totally agree.

##### Psychosocial Consequences and Coping 

Workaholism. Measured through a two-dimension scale adapted from Boada-Grau et al. [45]. The first dimension explores negative workaholism through 5 items (“I often find myself thinking about work, even when I want to rest for a while”; “I get bored and get restless during holidays when I do not have anything productive to do”; “I feel obliged to work hard even when it is not pleasant”; “It seems as if something inside me forces me to work hard”; I lose track of time when I am involved in a project”) (α = 0.74); while the second one explores positive workaholism through 2 items (“My work is so interesting that it often doesn’t feel like work”; “Most of the time my job is very pleasant”) (α = 0.73). All items were measured through the following question: “Please indicate how strongly you agree with the following statements”, on a Likert-type scale ranging from 1 = totally disagree to 7 = totally agree.

Job stress. Measured through a 4-item scale from Stanton et al. [58] (“Currently, in this work situation, to what extent do you feel the following way about your relationship to work?”; response items being: “under pressured”, “hectic”, “nerve-wracking”, “calm”) (α = 0.88), on a Likert-type scale ranging from 1 = not at all to 7 = totally.

Coping. Measured through 3 items taken from the positive thoughts dimension of Rodríguez-Marín et al. [59] (“I have promised myself to get something positive out of the situation”; “I have tried to see the positive side of things”; “I have considered the advantages of this situation”) (α = 0.83). All items were measured through the following question: “Please indicate how strongly you agree with the following statements regarding the way in which you have dealt with this situation”, on a Likert-type scale ranging from 1 = totally disagree to 7 = totally agree.

#### 3.1.4. Data Analysis

In order to find out whether there were differences in the variables studied according to gender, having dependents or working modality, 13 ANOVAs were carried out with three fixed 2 × 2 × 2 factors (work situation × gender × care) with the dependent variables described in the instruments section.

### 3.2. Results

First, descriptive results are presented, focusing on the results for the entire sample. Second, we present the results of the ANOVAS analyses.

Descriptive statistics (Table 5): regarding the socio-health situation, respondents scored moderately on fear of contagion and perceived economic threat. With regard to the new work organisation, almost a third of the population reported working longer hours, with this increased workload being particularly salient among teleworkers. In addition, those who teleworked rated the resources provided for teleworking as average, such as the organisation’s readiness to telework, and a third of them reported working with more productivity control measures. Besides, people who worked on-site rated safety in on-site work moderately. Regarding conciliation, workers experienced moderate work–family conflict and low family-work conflict. Finally, regarding psychosocial consequences and coping, low levels of negative workaholism and moderate positive workaholism were reported. Stress levels were reported to be medium-high, and workers stated the development of coping strategies during the lockdown situation. 

The results of the ANOVA analyses are presented below. The significant interactions of the three fixed factors (work situation × gender × care) ANOVAs conducted with the dependent variables are described. Tables S1 and S2 showing the results of the ANOVAs are presented in the Supplementary Material. Table 6 presents the descriptive statistics of the statistically significant two-way and three-way interactions.

Regarding socio-health situation, a triple interaction between gender, caregiving and working modality on fear of contagion (F (1, 239) = 4.102, *p* = 0.044, η2 = 0.017) is shown (Table S1, Supplementary Material). In particular, women with dependents who were working on-site felt the greatest fear of infection (M = 6.21; SD = 0.66) (Table 6).

Respecting conciliation variables, the association between caregiving and work–family conflict, F(1, 240) = 10.845, *p* = 0.001, η2 = 0.045, and family–work conflict F(1, 236) = 12.84, *p* < 0.001, η2 = 0.053, is identified (Table S2, Supplementary Material). Our results showed that people with dependents experienced higher levels of work–family conflict (M = 4.18; SD = 1.89) and family–work conflict (M = 3.29; SD = 1.90) than those without dependents (work–family conflict, M = 3.31; SD = 1.66; family–work–conflict, M = 2.42; SD = 1.43) (Table 5). As well, teleworkers showed higher levels of family–work conflict (M = 3; SD = 1.69) than those who worked on site (M = 2.06; SD = 1.21), F(1, 236) = 13.419, *p* < 0.001, η2 = 0.056 (Table 5 and Table S2, Supplementary Material). In addition, a double interaction was found between gender and work modality on family–work conflict (Table S2, Supplementary Material), F(1, 236) = 4.319, *p* = 0.039, η2 = 0.019. Specifically, teleworking women (M = 3.15; SD = 1.79) experienced higher levels of family-work conflict than teleworking men (M = 2.7; SD = 1.45) and people who worked on site (women, M = 2.00, SD = 1.12; men, M = 2.13, SD = 1.31) (Table 6).

Concerning psychosocial consequences, several interactions effects were found between gender and negative workaholism, F(1, 238) = 4.799, *p*= 0.029, η2 = 0.020, gender and job stress, F(1, 240) = 4.986, *p* = 0.027, η2 = 0.021, and gender and coping, F(1, 239) = 10.277, *p* = 0.002, η2 = 0.043 (Table S2, Supplementary Material). Particularly, women showed higher levels of negative workaholism (M = 2.96; SD = 1.11) and job stress (M = 4.58; SD = 1.44) than men (workaholism, M = 2.44; SD = 1.05; stress, M = 4.13; SD = 4.15), but more coping strategies (women, M = 5.31; SD = 1.15; men, M = 4.65; SD = 1.33) (Table 5). Finally, interactions between work situation and negative workaholism, F(1, 238) = 5.729, *p* = 0.017, η2 = 0.024, and work situation and positive workaholism, F(1, 241) = 9.171, *p*= 0.003, η2 = 0.038, were identified (Table S2, Supplementary Material). Specifically, teleworking people perceived more negative (M = 3.04; SD = 1.12) and positive (M = 4.18; SD = 1.31) workaholism than those who worked on site (negative workaholism, M = 2.35; SD = 0.98; positive workaholism, M = 3.22; SD = 1.48) (Table 5).

## 4. Discussion

Through two consecutive studies and taking into account a range of structural and psychosocial variables, this research aimed to arrive at a comprehensive and holistic understanding of workers’ experience and wellbeing during lockdown. The lockdown situation precipitated by the COVID-19 pandemic disrupted the daily lives of millions of workers [1]. While some were able to continue their work activity through teleworking, those working in sectors considered essential had to continue commuting to their workplaces. Others saw their work activity come to a standstill (in whole or in part); and, in some cases, people in the most vulnerable or precarious jobs began to suffer redundancies and unemployment almost from the beginning of the state of alarm.

All these labour changes have taken place in a climate of fear of the disease (Study 1 and 2). Moreover, results of Study 2 showed that women with dependents who were working on-site felt the greatest fear of infection. These results can be read in terms of gender-differentiated roles and the way in which women with caring responsibilities, compared to their male counterparts, may have developed greater experiences of guilt or assumption of responsibility in relation to leaving home (and private space) in the context of a health emergency.

Economic threat and future employment uncertainty were also present among participants in both studies, consistent with findings from previous pandemics [18,19]. From a gender perspective, in Study 1, some women made specific mention of the extraordinary vulnerability they would face in the future if schools remained closed, knowing that the need to reduce working hours to care for dependents would be an aggravating factor that would fall on them. These results are consistent with other studies finding that economic vulnerability is related to caring responsibilities [15], and the particular vulnerability of women caregivers [22]. However, the results of Study 2 showed no significant differences for this last group, which may indicate a lack of awareness of the vulnerability to which they are exposed, and the need to highlight this situation from a feminist perspective, that allows women to understand their situation from a more structural approach linked to their gender and labour market position.

Undoubtedly, the need to continue working and avoid economic collapse became a central issue for organisations, sometimes without considering that this was a global change affecting people’s whole lives. The forced improvisation of these changes meant in many cases, and especially among those who teleworked, an increase in time spent at work (Study 2) to support the adaptation process (Study 1), often in organisations without sufficient training for the necessary technological adaptation which took place in record time (Study 1 and 2).

In the case of teleworking, the results of this research show that organisations’ teleworking experience and the provided organisational resources were modestly valued (Study 1 and 2). While some access to virtual meeting platforms and training in their use was provided, the material resources were generally dependent on workers themselves. Indeed, the interest and training organisations have in information and communication technologies (ICT), as well as the training and resources provided to workers, has been noted in the literature as an indispensable condition for this adaptation [60].

Furthermore, scientific literature warns that teleworking is not compatible with an organisational culture based on control and presence, and that it requires a climate of trust and flexibility from supervisors [7]. However, some people made specific mention to the systematic distrust by organisations that workers would fulfil their obligations (Study 1), and a third of those who teleworked reported that additional mechanisms were put in place to monitor their work (Study 2). While these measures may be important to avoid overtime, testimonies suggest that these mechanisms were not aimed at protecting workers’ working conditions, but rather at ensuring productivity and achievement of organisational objectives (Study 1). 

In case of people working on-site, organisational resources available to deal with the possibility of contagion were rated as insufficient (Study 2). Among those who went to their workplaces in large companies, the testimonies report that extensive safety protocols were implemented as the virulence of the pandemic became known, even though it is said that they were not always as protected as they would have needed to be (Study 1). Workers in the smallest enterprises, which coincide with the most vulnerable jobs, seemed to report these shortcomings the most, being forced to work with greater exposure to infection (e.g., domestic workers) (Study 1). Not being afraid of becoming infected is essential to protect both the physical and mental wellbeing of workers [14,19]; and, in this sense, a key challenge for organisations in developing occupational risk prevention measures for their employees [61].

Undoubtedly, the work–life balance challenge has become a central issue in this pandemic, both in relation to caring for dependents and, in a broader sense, to work–life balance and work–life separation. In the case of teleworking, although the literature has outlined its advantages for work–life balance [28], the difficulties created by the permeability between work and family life [34], family interference [31] and family disputes caused by poor delimitation of spaces [32,33] were salient in an emergency context where workers had to exercise care-giving tasks without the support available in a normal situation (Study 1). In fact, results of Study 2 also showed that workers with care responsibilities expressed greater difficulties in balancing work with family and family with work.

Women teleworking also reported that they were particularly vulnerable to the inability to combine care with telework, which created stressful and tense situations for them (Study 1). In the case of men, the level of conflict experienced by teleworkers did not differ from that of men and women working on-site (Study 2). These results suggest that, during the lockdown period, family responsibility (with or without dependents) fell mainly on women, leading to more stressful situations for them [40,62]. Moreover, although negative workaholism levels were not high, women experienced greater negative workaholism compared to men (Study 2).

Moreover, the situation experienced during the pandemic is consistent with work and gender literature beyond crisis situations, stating that men and women telework under different conditions, with men having less work interference and more opportunities to focus on their job [37,38,39]. Furthermore, research outside crisis contexts shows that the causes of work–related stress experienced by men and women vary widely (i.e., for men, they are associated with job performance and professional development; while, for women, they are associated with double workloads) [62].

In addition, results of Study 2 showed that women developed more coping strategies in this situation, although this does not imply that they necessarily coped better with stress. In fact, consistent with the research of Rodriguez et al. [63], women expressed higher levels of stress compared to men (Study 2). Literature shows that women are indeed more likely to re-signify situations and try to learn from them when coping with difficult situations [54,64]. However, it is possible that, despite having more coping strategies, when work and family responsibilities exceed actual capacities, stress inevitably arises, making it impossible for women to respond to all demands. In fact, the pressure on women to act as superwomen who can do it all has intensified in recent years, forcing them to display a high capacity to cope with all life scenarios, in a context where the division of labour in the private sphere remains an unresolved challenge.

Moreover, teleworking was found to be associated with an increase in the number of hours spent at work (Studies 1 and 2). In fact, people who engage in telework tend to experience more negative workaholism [51,52], which is often related to increased isolation, lack of spatial differentiation and loss of control over work time. In addition, it was noted that organisations have tended to implement additional control measures on employees who have teleworked (Study 2), and testimonies also showed a difficulty in digital disconnection linked to the need to show that they were available to their colleagues and superiors (Study 1). Literature has shown that telework predicts a higher risk of increased working hours [65], and the lockdown context may have certainly reinforced this tendency. Thus, as the testimonies show, instead of implementing policies to avoid the occupational risks that may arise from this hyperconnectivity, organisations seem to have fostered a climate favourable to its emergence. Although teleworkers experienced the negative aspects of work during lockdown more intensely, they also experienced more positive workaholism. In this sense, compared to those who worked on-site, teleworkers were able to lose track of the time spent working and enjoy the activity, helping them to escape from the lockdown situation to a greater extent. Finally, it is to be welcomed that workers were able to develop coping strategies to deal with the emergency situation (Study 1 and 2). In coping with the consequences of lockdown and telework, employees showed that the ability to maintain better health and well-being was related to the possibility of establishing routines, limiting working time, and separating living spaces (Study 1) from working ones [53]. This recovery of living spaces outside of work was especially pointed out by those workers whose professional activity was paralysed during lockdown. In particular, the testimonies showed how some people took advantage of this period to reflect on the centrality of work in their lives in normal conditions, and the importance of reorganising life in terms of greater balance. 

There are several limitations that need to be highlighted in our study. An important limitation of this study was not being able to look further into respondents’ occupation and educational level. In addition to analysing the effects of working modality, gender and care, incorporating these two variables into the study could have helped to clarify some group differences. Its inclusion in the study would have been relevant, as we know that teleworking is a modality that is usually accessed by people with higher levels of responsibility and hierarchical position in organisations [66]. Although teleworking was widespread during lockdown, only a minority of workers had access to it [2]. This may have implied unequal access to this working modality for some sectors, but also increased difficulty in carrying out teleworking due to the greater novelty of this experience. Therefore, the work experience during the pandemic of different types of workers with different educational levels should be explored further in future studies.

Another limitation of our research is its cross-sectional design. The data were collected at a single point in time (between April and May 2020); thus, it was not possible to analyse the influence of the time elapsed on the contents analysed. No data were collected in relation to the start of the lockdown situation (15 March), nor the end of it (21 June). Presumably, the novelty and prolongation of the situation could have generated changes in the variables studied, which could not be analysed in this non-longitudinal study. Similarly, in the absence of pre-lockdown data, we do not know participants’ baseline levels, so we cannot assert that the observed effects are due to the pandemic situation, rather than to a pre-lockdown situation.

Moreover, another potential limitation of this study is the impossibility of generalising the results and consequences to the whole population. Conditioned by the lockdown situation, a non-probabilistic snowballing sampling strategy was used for the dissemination of surveys. Likewise, the surveys were disseminated through social networks, excluding people who did not have access to the Internet or social networks. The fact that the surveys were completed via a self-reported questionnaire may have also reduced the reliability of the study, in terms of the comprehensibility of the questions or the sincerity of responses to particularly sensitive questions (among others).

Finally, data prior to the pandemic indicate that the presence of men and women in telework was similar, both in Europe and Spain [67]. However, female teleworkers were over-represented in our sample. Although, the presence of women in telework has increased more during lockdown compared to men, both in Europe (13% women and 11.2% men) and in Spain (12.1% women and 9.9% men) [67], this does not fully explain the gender gap in our sample, which could be due to the non-probabilistic design of our study. 

## 5. Conclusions

Complementarily, results of both studies show that workers’ relationship with the COVID-19—work binomial during the lockdown period (perceived fears, changes in work organisation, family conciliation or psychosocial consequences and coping) was different depending on working modality, gender and whether they had dependents. Although the situation was faced by participants without serious consequences on their well-being at work, this research identified some variables in which a prolonged situation in these conditions could affect workers, providing clues that can be used to care for workers from an organisational health perspective.

Gender emerged as an essential variable in understanding this experience. Although women have been able to develop more coping strategies in this situation, results revealed greater stress among them, more conflicts arising from conciliation demands, a greater fear of illness among those with dependents, and even, in some cases, greater warning of risks because of their precarious work situation. This calls for more research to further study how this situation, if prolonged over time, may affect women from a comparative perspective. It is also worth highlighting the importance of organisational role in the well-being of employees, and how this will be related to their capacity to respond or not to the changes that arise in these crisis contexts (capacity to adapt, establishment of health protocols, support for teleworking, conciliation measures, etc.). 

Finally, if organisations want to take advantage of this situation to extend telework in those jobs where possible, the data from this study confirm the relevance of accompanying it with changes in the organisational culture, replacing control culture with a culture of trust; as well as respecting the specific legislation in this area, in order to tackle the problems of hyperconnectivity, workaholism and the impossibility of combining work and family life, which were reflected in both studies.

## Figures and Tables

**Table 1:** Theme 1 developed from thematic analysis in Study 1: “work and socio-health situation”.

Sub-Themes and Codes	Quotes
Work situation	
Going to the worksite	“My work has been necessary from the very beginning; I have not been obliged to stay at home.” (Woman, 33, nursing, no dependent children, working on-site)
Teleworking without reduced activity/pay	“So far it has not affected my salary and working hours.” (Woman, 45, NGO ^1^, foreign, with dependent children, teleworking); “As I work for the public administration, I have continued to telework for the same pay.” (Woman, 39, university lecturer, no dependent children, teleworking).
Teleworking with reduced activity/pay	“My normal working day is 40 h and with the COVID situation it was unilaterally established by the administration that we would be counted for 35 h.” (Woman, 46, public administration, no dependent children, teleworking).
Stalled activity	“Now things are at a standstill and we workers don’t know anything.” (Woman, 60, private company, with dependent children, ERTE ^2^).
Layoffs	“I have been out of work since the lockdown was decreed. The person who used to give me work told me that he is not going to employ me again.” (Woman, 26, domestic work, foreign, with dependent children, unemployed).
Socio-health situation	
Fear of contagion	“The biggest stress has been leaving home and going to work when there was such a high risk of infection. Touching doors, entering places without knowing if it was sufficiently disinfected” (Woman, 48, private company, no dependent children, working on-site); “Although we are at home, we are very afraid. The media are saying: contagions, contagions.” (Woman, 45, NGO ^1^, foreign, with dependent children, teleworking).
Economic threat	“We have not kept the customers and we have not generated any profit: the company has lost a lot of money. There is fear for the future” (Woman, 24, private company, no dependent children, teleworking).

^1^ NGO: non-governmental organization. ^2^ ERTE: temporary employment regulation file.

**Table 2:** Theme 2 developed from thematic analysis in Study 1: “consequences on work organisation and resources available for change”.

Sub-Themes and Codes	Quotes
Changes in work organisation	
Resistance to change	“Everything has been about trying to continue with normality. It was hard to assimilate that not everything was possible.” (Man, 34, education, no dependent children, ERTE ^1^); “Until the last few weeks I have not seen a reaction from my bosses. They have not reacted.” (Woman, 46, public administration, no dependent children, teleworking).
Mistrust	“There was a lot of surveillance, a lot of mistrust, a lot of intense control.” (Woman, 58, university lecturer, no dependent children, teleworking).
Responsibility	“We had to build the urgencies from scratch; a responsibility that was not ours to bear.” (Woman, 23, nursing, no dependent children, working on-site).
Adaptation	“A big stress peak in the first few weeks due to the novelty of everything.” (Woman, 46, public administration, no dependent children, teleworking); “It has been a further burden of adapting to new protocols and new areas of work.” (Woman, 23, nursing, no dependent children, working on-site).
Difficulties	“There are processes that have been more difficult to transfer to teleworking, solving problems that are easier face-to-face.” (Man, 41, public administration, no dependent children, teleworking).
Resources	
Sanitary measures	“Masks, distance, etc. The manager asked us how we were feeling (headache, fever). Quite a lot of control” (Man, 25, private company, no dependent children, working on-site); “They gave us a mask for 5 days. I take care of elderly people: cleaning them, changing nappies, everything. Those of us who work in homes have the right to be tested, it’s the least we deserve.” (Woman, 46, foreign, no dependent children, domestic work, working on-site).
Material resources	“No specific material support for work (computers) has been provided, electricity costs have not been covered, and the right to digital disconnection has not been guaranteed.” (Man, 34, education, no dependent children, ERTE ^1^).
Training	“We had two days of very quick and basic training to get to know the platform and to be able to telework.” (Woman, 58, SME ^2^, with dependent children, teleworking).

^1^ ERTE: temporary employment regulation file. ^2^ SME: small and medium-sized enterprises.

**Table 3:** Theme 3 developed from thematic analysis in Study 1: “work–life balance management and centrality of life in the lockdown context”.

Sub-Themes and Codes	Quotes
Conciliation	
Work–life	“I usually start my workday when I arrive at the office and try to finish it when I leave. Now I have mixed everything up” (Man, 31, teacher, no dependent children)
Family–work	“The child wanted me to play with him and as soon as I got on the computer he started to cry, he demanded a lot from me. I had all my papers all over the place. I had no space for myself, and the child made it more difficult for me.” (Woman, 36, teacher, with dependent children).
Work–family	“I was stressed because I didn’t look after my children well. We had to start abusing TV, which I don’t like at all; a lot of guilt and stress.” (Woman, 36, NGO ^1^, with dependent children); “Taking hours away from my family. If before I was scolded for working too much, now even more so because there are no schedules.” (Woman, 58, SME ^2^, with dependent children).
Care support	“While my partner was at home, I was able to work, but when he went back to work, it was hell.” (Woman, 36, NGO ^1^, with dependent children).
Accentuation of gender roles	“Combining my work with school follow-up, which my partner is not in charge of; he only has to tell me his timetable so that I can organise it. If there is no school in September, I will have to reduce my working hours: we women are condemned to live a doomed life.” (Woman, 36, NGO ^1^, with dependent children).
Addiction/life centrality	
Work centrality	“Everything has been about work; I don’t have anyone else; I am single and live alone. I dedicate everything to work” (Man, 41, Public Administration, no dependent children).
Hyperconnectivity	“It was some kind of nervousness, that they know that I am at my workplace, attentive to communications and to everything.” (Woman, 45, private company, no dependent children).
Disconnection and rest	“Having time to do what I like: realising the importance of working to live and not living to work.” (Man, 34, self-employed, no dependent children).

^1^ NGO: non-governmental organization. ^2^ SME: small and medium-sized enterprises.

**Table 4:** Theme 4 developed from thematic analysis in Study 1: “psychosocial consequences and coping with the situation”.

Sub-Themes and Codes	Quotes
Psychosocial consequences and coping	
Isolation	“We have locked ourselves at home and until this month, we have had no meetings to support us.” (Woman, 36, NGO ^1^, with dependent children); “I have missed the social relationship with colleagues: that is something that a teleconference cannot replace.” (Man, 57, NGO ^1^, with dependent children).
Technological exhaustion	“For me, everything online is very tiring. When we used to do video conferences for work or with a friend, I end up getting exhausted with it.” (Woman, 48, private company, no dependent children).
Physical and psychological exhaustion	“At home I don’t have the comfortable chair I have at work, so the whole issue of prevention of occupational hazards, back pains, etc.” (Woman, 46, Public Administration, no dependent children); “Working five days a week is not the same as resting one day out of eight. It’s a job that takes a lot out of you physically and psychologically.” (Woman, 33, nursing, no dependent children).
Lack of ventilation spaces	“Yes, I have felt much more tense, because if you leave work and go for a walk, you get some fresh air. However, not being able to go out and freshen up, I have been more irritable.” (Woman, 48, private company, no dependent children).
Stress and burnout	“There have been times when the situation has overwhelmed me, and I have exploded. I have needed psychological help” (Woman, 45, NGO ^1^, foreigner, with dependent children).
Coping	“I think I have been able to see the positive side of it. It has allowed me to develop professionally: once you get over those fears of videoconferencing, it’s an enriching experience.” (Woman, 46, Public Administration, no dependent children, teleworking).

^1^ NGO: non-governmental organization.

**Table 5:** Descriptive statistics for study variables in Study 2, by gender, care (having dependents or not), and work situation.

Variables		Gender		Care (Dependents)		Work Situation	
	Total	Woman	Man	Yes	No	Telework	On-Site
	M (SD)	M (SD)	M (SD)	M (SD)	M (SD)	M (SD)	M (SD)
Socio-health situation							
Fear of contagion	4.93 (1.52)	4.99 (1.51)	4.85 (1.53)	5.26 (1.45)	4.85 (1.53)	4.75 (1.42)	5.18 (1.61)
Perceived economic threat	4.65 (1.17)	4.72 (1.14)	4.55 (1.2)	4.9 (1.1)	4.59 (1.18)	4.54 (1.14)	4.8 (1.9)
New work organisation							
Hours worked	2.04 (0.71)	2.09 (0.75)	1.95 (0.69)	2.14 (0.74)	2 (0.72)	2.07 (0.78)	1.98 (0.64)
Less	23.6%	23.6%	26.3%	19.6%	26.4%	25.5%	21.2%
Same	48.5%	43.8%	52.5%	47.1%	47.7%	40%	59.1%
More	27.9%	32.6%	21.2%	33.3%	25.9%	34.5%	19.7%
Resources provided for teleworking	3.83 (1.52)	3.72 (1.5)	4.05 (1.56)	3.65 (1.61)	3.88 (1.5)	3.84 (1.52)	
Organisation’s readiness to telework	4.26 (1.6)	4.33 (1.53)	4.12 (1.73)	4.17 (1.65)	4.28 (1.59)	4.26 (1.6)	
Safety in on-site work	3.99 (1.77)	4.1 (1.77)	3.88 (1.77)	3.55 (1.93)	4.1 (1.72)		3.99 (1.77)
Productivity control measures	32.7% YES	39.32% YES	28.57% YES	48.27% YES	31.25% YES	32.7% YES	
Conciliation							
Work-family conflict	3.49 (1.74)	3.63 (1.82)	3.27 (1.61)	4.18 (1.89)	3.31 (1.66)	3.63 (1.65)	3.29 (1.85)
Family-work conflict	2.59 (1.57)	2.73 (1.67)	2.4 (1.4)	3.3 (1.9)	2.42 (1.43)	3 (1.69)	2.06 (1.21)
Psychosocial consequences and coping							
Negative workaholism	2.77 (1.1)	2.96 (1.11)	2.44 (1.05)	2.79 (1.23)	2.74 (1.09)	3.04 (1.12)	2.35 (.98)
Positive workaholism	3.77 (1.47)	3.91 (1.34)	3.58 (1.61)	3.66 (1.47)	3.8 (1.46)	4.18 (1.31)	3.22 (1.48)
Coping	5 (1.26)	5.31 (1.15)	4.65 (1.33)	5.04 (1.15)	5.05 (1.29)	5.13 (1.19)	4.93 (1.35)
Job stress	4.4 (1.46)	4.58 (1.44)	4.13 (1.45)	4.65 (1.75)	4.33 (1.37)	4.26 (1.42)	4.58 (1.48)

**Table 6:** Descriptive statistics for statistically significant two-way and three-way interactions.

Dependent Variable	Work Situation	Care	Man	Woman	Total Row
Family–work conflict	Teleworking	Yes	3.48 (1.33)	4.00 (2.30)	3.83 (2.01)
		No	2.50 (1.42)	2.94 (1.59)	2.79 (1.54)
		Total	2.70 (1.45)	3.15 (1.79)	3.00 (1.69)
	Working on site	Yes	3.29 (2.00)	2.11 (0.90)	2.58 (1.52)
		No	1.90 (1.03)	1.97 (1.18)	1.93 (1.10)
		Total	2.13 (1.31)	2.00 (1.12)	2.06 (1.21)
Fear of contagion	Teleworking	Yes	5.28 (1.20)	4.82 (1.51)	4.97 (1.42)
		No	4.50 (1.54)	4.80 (1.36)	4.69 (1.42)
		Total	4.65 (1.5)	4.8 (1.38)	4.75 (1.42)
	Working on site	Yes	4.94 (1.93)	6.21 (0.66)	5.70 (1.43)
		No	5.07 (1.48)	5.04 (1.80)	5.06 (1.64)
		Total	5.05 (1.54)	5.31 (1.68)	5.18 (1.61)

## Data Availability

The data presented in this study are available on request from the corresponding author. The data are not publicly available due to is personal information.

## Supplementary Materials

The following are available online at https://www.mdpi.com/article/10.3390/ijerph182212087/s1, Table S1: Three fixed 2 × 2 × 2 factors (gender × care × work situation) analysis of variance (ANOVA) in socio-health situation and new work organisation, Table S2: Three fixed 2 × 2 × 2 factors (gender × care × work situation) analysis of variance (ANOVA) in conciliation and psychosocial consequences and coping.
