# Supplementary material for: (Tele)Work and Care during Lockdown: Labour and Socio-Familial Restructuring in Times of COVID-19
Source: Int J Environ Res Public Health. 2021 Nov 17;18(22):12087. doi: 10.3390/ijerph182212087 (PMC8620492; doi:10.3390/ijerph182212087)
Supplement: Supplementary file 1 [file ijerph-18-12087-s001.zip › ijerph-1408959-supplementary.pdf]

Table S1. Three fixed 2 × 2 × 2 factors (gender × care × work situation) analysis of variance (ANOVA) in socio-health situation and new work organisation.

	**Socio-Health Situation**						**New Work Organisation**							
	**Fear of Contagion**		**Perceived Economic Threat**		**Hours Worked**		**Resources for Teleworking**		**Org. Readiness to Telework**		**Safety in On-Site Work**		**Productivity Control Measures**	
	**F (1, 239)**	**η^2**	**F (1, 240)**	**η^2**	**F (1, 243)**	**η^2**	**F (1, 135)**	**η^2**	**F (1, 140)**	**η^2**	**F (1, 102)**	**η^2**	**F (1, 138)**	**η^2**
Gender	1.129	0.005	0.130	0.001	1.307	0.006	0.985	0.007	0.393	0.003	0.012	0.001	1.831	0.013
Care	3.296	0.014	3.545	0.015	1.169	0.005	0.350	0.003	0.122	0.001	1.245	0.013	1.483	0.011
Work situation	3.353	0.014	2.041	0.009	1.386	0.006								
Gender * Care	0.302	0.001	1.347	0.006	0.021	0.000	0.008	0.001	0.004	0.001	1.215	0.12	0.452	0.003
Gender * Work situation	1.882	0.008	0.038	0.001	1.400	0.006								
Care * Work situation	0.051	0.001	0.010	0.001	0.625	0.003								
Gender * Care * Work situation	4.102 *	0.017	0.007	0.001	2.557	0.011								

Notes. * $p < 0.05$.

Table S2. Three fixed 2 × 2 × 2 factors (gender × care × work situation) analysis of variance (ANOVA) in conciliation and psychosocial consequences and coping.

	**Conciliation**				**Psychosocial Consequences and Coping**							
	**Work–Family Conflict**		**Family–Work Conflict**		**Negative Workaholism**		**Positive Workaholism**		**Coping**		**Job Stress**	
	**F (1, 240)**	**η^2**	**F (1, 236)**	**η^2**	**F (1, 238)**	**η^2**	**F (1, 241)**	**η^2**	**F (1, 239)**	**η^2**	**F (1, 240)**	**η^2**
Gender	0.661	0.003	0.028	0.001	4.799 *	0.020	2.551	0.011	10.277 **	0.043	4.986 *	0.021
Care	10.845*	0.045	12.840 **	0.053	0.040	0.000	0.897	0.004	0.271	0.001	1.071	0.005
Work situation	0.410	0.002	13.419 **	0.056	5.729 *	0.024	9.171 **	.038	0.610	0.003	0.682	0.003
Gender * Care	0.010	0.001	1.350	0.006	0.021	0.000	1.398	0.006	0.036	0.001	0.071	0.000
Gender * Work situation	0.080	0.001	4.319 *	0.019	0.963	0.004	0.431	0.002	0.300	0.001	1.626	0.007
Care * Work situation	0.362	0.002	0.259	0.001	2.376	0.010	2.226	0.009	0.329	0.001	0.924	0.004
Gender * Care * Work situation	1.992	0.009	1.775	0.008	0.929	0.004	0.939	0.004	0.843	0.004	0.755	0.003

Notes. * $p < 0.05$. ** $p < 0.01$.
